# Meiofauna promotes litter decomposition in stream ecosystems depending on leaf species

**DOI:** 10.1002/ece3.6610

**Published:** 2020-08-03

**Authors:** Fang Wang, Dunmei Lin, Wei Li, Pengpeng Dou, Le Han, Mingfen Huang, Shenhua Qian, Jingmei Yao

**Affiliations:** ^1^ Key Laboratory of the Three Gorges Reservoir Region's Eco‐Environment Ministry of Education Chongqing University Chongqing China; ^2^ College of Environment and Ecology Chongqing University Chongqing China

**Keywords:** litter decomposition process, macrofauna, meiofauna, microbes, trophic complexity

## Abstract

Litter decomposition, a fundamental process of nutrient cycling and energy flow in freshwater ecosystems, is driven by a diverse array of decomposers. As an important component of the heterotrophic food web, meiofauna can provide a trophic link between leaf‐associated microbes (*i.e*., bacteria and fungi)/plant detritus and macroinvertebrates, though their contribution to litter decomposition is not well understood. To investigate the role of different decomposer communities in litter decomposition, especially meiofauna, we compared the litter decomposition of three leaf species with different lignin to nitrogen ratios in litter bags with different mesh sizes (0.05, 0.25, and 2 mm) in a forested stream, in China for 78 days. The meiofauna significantly enhanced the decomposition of leaves of high‐and medium‐ quality, while decreasing (negative effect) or increasing (positive effect) the fungal biomass and diversity. Macrofauna and meiofauna together contributed to the decomposition of low‐quality leaf species. The presence of meiofauna and macrofauna triggered different aspects of the microbial community, with their effects on litter decomposition varying as a function of leaf quality. This study reveals that the meiofauna increased the trophic complexity and modulated their interactions with microbes, highlighting the important yet underestimated role of meiofauna in detritus‐based ecosystems.

## Graphical abstract

Effects of meiofauna on litter decomposition were estimated. Increasing trophic complexity leads to increased decomposition rate. Meiofauna significantly affected fungal biomass and diversity, and the biotic interactions were leaf species dependent.

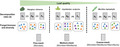

## 1 INTRODUCTION

Leaf litter decomposition, by providing an important source of energy for aquatic biota, has been recognized as a critical process for the functioning of forested streams (Chauvet et al., 2016; Gessner & Chauvet, 2002). Litter decomposition is driven by the biological activities of bacteria, fungi, and detritivorous macroinvertebrates referred to as shredders (Gaudes, Artigas, Romaní, Sabater, & Muñoz, 2009; Villanueva, Albariño, & Canhoto, 2012; Wymore et al., 2016). Macroinvertebrates, by consuming leaves and colonized fungi, significantly affect litter decomposition (Santonja, Pellan, & Piscart, 2018; Villanueva et al., 2012). Microbes, especially fungi, are responsible for a significant fraction of litter decomposition, and their colonization on leaves improves the palatability of litter for shredding macroinvertebrates by increasing the mycelial biomass and enzymatic metabolism (Bärlocher, 2005; Cornut, Ferreira, Gonçalves, Chauvet, & Canhoto, 2015).

While the contributions of fungi and shredding macroinvertebrates in litter decomposition have been well documented, the role of the meiofauna is rarely reported in the decomposition process (Palmer, 1997; Swan & Palmer, 2000). Indeed, growing evidence suggests the potential importance of meiofauna in litter decomposition (Ágoston‐Szabó, Schöll, Kiss, & Dinka, 2016; Chambord, Tackx, Chauvet, Escolar, & Colas, 2017; Perlmutter & Meyer, 1991). Meiofauna, regarded as an important component of the benthic community, include diverse species that are associated with leaf detritus (Hakenkamp & Morin, 2000). They show extremely high densities and high population turnover rates, so that collectively, their secondary production (and their energetic needs) are comparable or even higher than those of the macrofauna (Majdi, Threis, & Traunspurger, 2016; Reiss & Schmid‐Araya, 2010). The meiofauna can provide a key trophic link between leaf‐associated microbes (i.e., bacteria and fungi)/plant detritus and macroinvertebrates (Majdi & Traunspurger, 2017; Schmid‐Araya, Schmid, Tod, & Esteban, 2016).

Meiofauna may significantly contribute to litter decomposition in the following ways. First, they can act as microdetritivores by directly consuming leaves. For example, harpacticoid copepods effectively remove organic matter, fungi, and bacteria that accumulate in the debris (Perlmutter & Meyer, 1991). Meanwhile, the meiofauna can also indirectly affect litter decomposition by changing the microbial decomposition of leaves. Some meiofauna, such as rotifers and nematodes, feed on the associated biofilms. The grazing may alter the microbial abundance and/or the community structure, increasing or decreasing the microbial decomposition activity of microbes (Chambord et al., 2017). Also, the bioturbation effect of meiofauna can indirectly affect the process of microbial decomposition by changing the microenvironment associated with decomposition (oxygen, organic concentration, etc.) (Bonaglia, Nascimento, Bartoli, Klawonn, & Brüchert, 2014; Nascimento, Näslund, & Elmgren, 2012). Moreover, the meiofauna is subject to a top‐down control from macrofauna. Some meiofauna may compete with macrofauna for food or probably improve the palatability of detritus for macrofauna (Chambord et al., 2017; Perlmutter & Meyer, 1991; Ptatscheck, Brüchner‐Hüttemann, Kreuzinger‐Janik, Weber, & Traunspurger, 2020).

The common method used to assess the litter decomposition process is based on litter bags of two mesh sizes (i.e., coarse and fine mesh to separate the decomposition of macrofauna and microbes; Graça, Barlocher, & Gessner, 2005), which may blur the contributions of microbes and meiofauna. To date, their interaction with microbial communities and the contribution of meiofauna to the litter decomposition have not yet been elucidated. Moreover, the chemical characteristics of leaf litter (e.g., initial N and lignin contents and the lignin:N ratio) are known to affect microbial and invertebrate colonization and activity, consequently affecting litter decomposition (Alonso, González‐Muñoz, & Castro‐Díez, 2010; Sales, Gonçalves, Dahora, & Medeiros, 2014). Invertebrates can significantly consume the high‐quality leaf litter, whereas the decomposition of low‐quality leaf litter may be under the synthetic action of microbes and invertebrates (Raposeiro, Ferreira, Gea, & Gonçalves, 2018; Santonja et al., 2018). Thus, leaf chemical qualities may modify the contributions of meiofauna and other decomposers to the decomposition process, though the exact mechanisms remain unclear.

This study aimed to assess the effects of different decomposer communities, especially meiofauna, on the decomposition of leaves with varied chemical qualities. Three different mesh sizes were used in the decomposition bags to establish a gradient of increasing trophic and community complexity: (a) microbes; (b) microbes and meiofauna; (c) microbes, meiofauna, and macrofauna. We hypothesized that the presence of meiofauna and macrofauna should trigger different effects on the community structure of microbes, and these effects on litter decomposition should vary as a function of leaf quality. Specifically, for the high‐quality leaf species, meiofauna should promote the litter decomposition mainly through direct consumption as macrofauna. With the decreasing quality of leaves, the direct consumption of meiofauna and their interaction with microbes together should promote the litter decomposition.

## 2 METHODS

### 2.1 Study site and selected species

**FIGURE 1:**
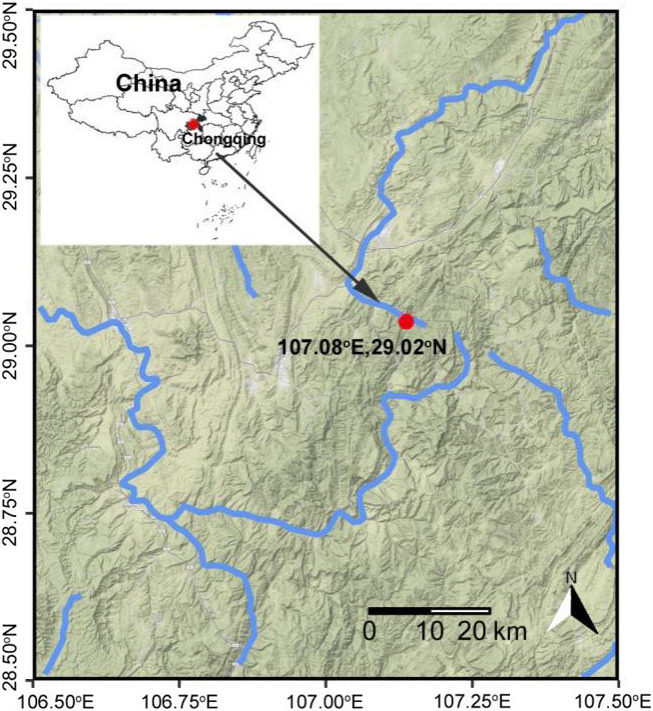
The study site (red spot) located in Jinfo Mountain, south‐western China

This study was conducted in a forested headwater stream, located in Jinfo Mountain National Nature Reserve (28°46′‐29°38′ N, 106°54′‐107°27′ E, altitude about 1,000 m), Chongqing, south‐western China (Figure 1). This region has subtropical humid monsoon climate, with a mean annual temperature of 14.5°C and a mean annual precipitation of 1,395.5 mm. The surrounding forest composed of mixed typical evergreen and deciduous broad‐leaved species, mainly including *Neolitsea confertifolia* (Hemsl.), *Chimonobambusa utilis* (Keng) P. C. Keng, *Cinnamomum subavenium* Miq., *Liquidambar acalycina* H. T. Chang, *Cornus controversa* Hemsl.,* Alangium chinense* (Lour.) Harms., *Fagus longipetiolata* Seem., and *Machilus leptophylla* Hand.‐Mazz (Qian et al., 2016). Leaves from three riparian forest species were selected for the field experiment that considered leaf chemical characteristics and their dominance at the study site (i.e., *Alangium chinense*, *Liquidambar acalycina*, and *Machilus leptophylla*). The leaf characteristics were determined from litter material of the original batches, including carbon and nitrogen (TOC/TN auto‐analyzer, Shimadzu TOC‐L CPH, TNM‐1, Japan), phosphorus (molybdenum blue photometric method after basic digestion with sulfuric acid and 30% hydrogen peroxide, Jones, 2001), lignin (acid‐detergent fiber method, Graça et al., 2005), toughness (tensile force required to tear the blade in unit width using a tensiometer (ZP‐50, Aigu, HongKong, China; Pérez‐Harguindeguy et al., 2000).

### 2.2 Water characteristics

The physico‐chemical characteristics of the stream water were measured at each sampling date with litter bags retrieved during the experimental period. The temperature, pH, conductivity, and dissolved oxygen of the stream water were measured in situ using a multi‐parametric sensor (YSI Professional plus, Yellow Spring, USA) at each sampling date. The ammonium (NH_4_^+^), nitrate (NO_3_^−^), and soluble reactive phosphorous (${\text{PO}}_{4}^{3-}$) from filtered water samples were analyzed by a spectrophotometer (UV2600; Shimadzu) in the laboratory following the standard methods (APHA, 2005). Water temperature decreased from 14.7°C in autumn to 5.1°C in winter during the decomposition experiment. During the experimental period, none of the measured physico‐chemical characteristics of the stream water, except temperature, differed significantly among the sampling dates (*p* > .05; Table 1).

**TABLE 1:** Physico‐chemical parameters of stream water during litter decay (mean (min–max), *n* = 12)

	Water temperature (°C)	Dissolved oxygen (mg/L)	pH	Conductivity (μS/cm)	NH_4_^+^ (mg/L)	NO_3_^‐^ (mg/L)	PO_4_^3−^ (mg/L)
Mean (min–max)	10.2 (5.1–14.7)	9.63 (9.12–10.48)	7.89 (7.44–8.10)	178.9 (161.6–196.7)	0.26 (0.12–0.40)	0.85 (0.10–1.93)	0.042 (0.031–0.057)

### 2.3 Experimental design

The litter decomposition experiment was carried out between October 2017 and January 2018. Leaves were collected immediately after abscission in September 2017 and dried at 60°C for 48 hr. Three grams (3 ± 0.1 g) of each leaf species was enclosed in the bags (15 × 10 cm). The litter bags were sprayed with deionized water after being weighed to prevent leaf breakage during handling and transport to the field. Three different mesh sizes (0.05, 0.25, and 2 mm) were chosen to restrict organisms of different body size: fine mesh (0.05 mm) mainly allowed access for microbes. Although small nematodes and rotifers can pass through 0.05 mm mesh, the fine mesh can restrain access to most meiobenthos (like chironomids, copepods, and oligochaetes). The medium mesh (0.25 mm) allowed access for microbes and meiofauna. The entire decomposer community, including microbes, meiofauna, and macrofauna, can enter the coarse mesh (2 mm). The increasing mesh size was expected to induce an increase in the faunal community complexity and trophic complexity (Bradford, Tordoff, Eggers, Jones, & Newington, 2002). With this approach, the microbial contribution to decomposition was assumed to correspond to the mass loss in fine‐mesh bags, and the difference in mass loss between different sized meshes was interpreted to be equivalent to invertebrate‐mediated decomposition.

A total of 108 litter bags (3 species × 3 mesh sizes × 4 replicates × 3 times) were tied to nylon lines that were anchored randomly to the stream bed using house bricks in shallow riffles. Water depth at base flow was 5–30 cm at the place where the litter bags were exposed. Four replicates for the treatments in species and mesh size were retrieved on days 21, 47, and 78. The three collection dates were selected, according to the early, middle, and late stage for the species that decomposed fastest. Each litter bag was sealed individually in one plastic bag when they were still immersed, before being transferred in a cool box to the laboratory. The leaf from each bag was gently rinsed with distilled water to remove other material and then put into vacuum freeze‐dryer. From each bag, a subsample (weighing 0.6 g) was taken for microbial measurement and stored at −80°C for further analyze. The weight of the subsample was taken into consideration when the litter decomposition rate was calculated. The remaining litter material was dried (60°C for 48 hr) and weighed to determine the final dry mass.

### 2.4 Fungal biomass

The fungal biomass on the leaves was estimated by ergosterol concentration of freeze‐dried, pulverized leaf subsamples (Graça et al., 2005). Lipid extraction was carried out in KOH methanol at 80°C for 30 min. Extracted lipids were purified using solid‐phase extraction (SPE) cartridges (Supelclean™ LC‐18 SPE Tubes 500 mg), and ergosterol was eluted with isopropanol. The ergosterol concentration was quantified with high‐pressure liquid chromatography (HPLC, Agilent 1100), with 100% methanol as the mobile phase (1.4 ml/min, column temperature 33°C, measuring absorbance at 282 nm). Finally, the results were expressed as mg of fungal biomass per gram of leaf litter dry mass (DM). The data were converted to fungal biomass by the average conversion factor of 5.5 mg of ergosterol per gram of fungi (Gessnert & Chauvet, 1993).

### 2.5 Microbial communities

Microbial DNA was extracted from freeze‐dried, pulverized leaf subsamples (the second sampling on day 47) using a FastDNA^®^ Spin Kit for Soil (MP) according to manufacturer's guidelines. The final DNA concentration and purification were confirmed by NanoDrop 2000 UV‐vis spectrophotometer (Thermo Scientific), and DNA quality was checked by 1% agarose gel electrophoresis. The V4 hypervariable region of the bacteria 16S rRNA gene was amplified using primers 515F (5′‐GTGCCAGCMGCCGCGG‐3′) and 806R (5′‐GGACTACHVGGG TWTCTAAT‐3′; Caporaso et al., 2011). The ITS1 hypervariable region of the fungal ITS gene was amplified using primers ITS1F (5′‐CTTGGTCATTTAGAG GAAGTAA‐3′) and ITS2R (5′‐GCTGCGTTCTTCATCGATGC‐3′; Tolkkinen et al., 2015).

#### 2.5.1 Microbial abundance

Fungal and bacterial abundances were assessed with real‐time quantitative PCR (qPCR). Standard curves were analyzed by 10‐fold serial dilutions of a plasmid (pGEM‐T) containing the targeted gene inserts for the ITS and 16S rRNA gene. The qPCR reactions were performed in duplicate as 20 μl mixtures, each containing 16.5 μl ChamQ SYBR Color qPCR Master Mix, 0.8 μl of each forward and reverse primer, and 2 μl Template (DNA). Amplification was conducted on an ABI Prism^®^ 7500 Real‐Time PCR system (Applied Biosystems) with the following settings: initial denaturation at 95°C for 5 min, followed by 40 cycles, consisting of denaturation at 95°C for 5 s, annealing at 58°C or 60°C for 30 s separately for bacterial and fungal genes, and extension was at 72°C for 40 s. Finally, fungal and bacterial gene copy numbers were calculated according to the standard curves.

#### 2.5.2 Microbial community structure

Fungal and bacterial communities were estimated using high‐throughput sequencing methods. Amplicons were pooled in equimolar amounts and paired‐end sequenced on the Illumina MiSeq platform (Illumina) at Majorbio Bio‐Pharm Technology Co. Ltd. Raw fastq files were demultiplexed, quality‐filtered by Trimmomatic and merged by FLASH according to the criteria, detailed in Qin et al. (2018). Operational taxonomic units (OTUs) were clustered based on a 97% identity cut‐off using UPARSE (version 7.1 http://drive5.com/uparse/), and chimeric sequences were identified and removed using UCHIME. The taxonomy of each 16S/ITS gene sequence was analyzed by the RDP Classifier algorithm (http://rdp.cme.msu.edu/) against the Unite database for fungal sequences and the Silva database for bacterial sequences. Unclassified OTUs were annotated using basic local alignment search tool (BLAST) searches of National Center for Biotechnology Information (NCBI), USA GenBank's nonredundant nucleotide database. The naming of OTUs was based on the best BLAST hits.

### 2.6 Benthic meiofauna and macrofauna

Benthic meiofauna and macrofauna were sampled at the time when litter bags were put in and then retrieved. At each time, three samples covering most microhabitats present in riffles in the study reach were collected for macrofauna and meiofauna using two successive nets with mesh sizes of 250 and 50 µm. Samples were preserved in 75% ethanol and transferred to the laboratory. Macrofauna and meiofauna were sorted and identified to the lowest possible taxonomic level when possible (mainly to genus or species level), except for Oligochaeta (Class level), Diptera (Family or subfamily level), Copepods (Subclass level), Nematodes (Phylum level), and Ostracoda (Class level). Functional feeding groups were assigned according to Majdi Traunspurger Richardson and Lecerf (2015), Morse, Yang, and Tian (1994), and Tachet, Richoux, Bournaud, and Usseglio‐Polatera (2002).

### 2.7 Statistical analyses

Litter decomposition rates (*k*) were determined using the negative exponential model:

$$M_{t}=M_{o}e^{-kt}$$

where M_t_ is the remaining mass at time *t* (in days), and *M*_o_ is the initial mass (Graça et al., 2005). The leaf mass remaining (%), denoted as *R*, was calculated according to the formula:

$$R=\left(\frac{M_{t}}{M_{o}}\right)\times 100\%.$$

The effect or relative contribution of microbes, meiofauna, and macrofauna, denoted as *E*, to leaf mass loss denoted as *L*, was quantified based on the following formulas (Chen & Wang, 2018; Seastedt, Todd, & James, 1987):

$$E\left(\text{microbes}\right)=\left(\frac{L\left(\text{microbes}\right)}{L\left(\text{total}\right)}\right)\times 100\%$$

$$E\left(\text{meiofauna}\right)=\left(\frac{L\left(\text{meiofauna}\right)}{L\left(\text{total}\right)}\right)\times 100\%$$

$$E\left(\text{macrofauna}\right)=\left(\frac{L\left(\text{macrofauna}\right)}{L\left(\text{total}\right)}\right)\times 100\%$$

where *L* (microbes) is the mass loss from fine‐mesh bags; *L* (meiofauna) is the difference in mass loss between the fine‐ and medium‐mesh bags; *L* (macrofauna) is the difference in mass loss between the medium‐ and coarse‐mesh bags; and *L* (total) is the mass loss in the coarse‐mesh bags. Microbial Shannon diversity indexes (*H*′) were calculated based on the number of OTUs using the online Majorbio cloud platform (www.majorbio.com).

A three‐way ANOVA was conducted to determine the effect of leaf species, mesh size, time, and their interactions on leaf mass remaining and fungal biomass. A two‐way ANOVA was performed to determine the effect of leaf species and mesh size on litter decomposition rates (*k*). A one‐way ANOVA was applied to compare differences for litter decay (litter decomposition rates and mass remaining) and microbial parameters (fungal biomass, and fungal and bacterial abundance) among mesh size at each time for each species. LSD post hoc tests were performed when the ANOVAs detected a significant difference. For all parametric analyses, normality and homogeneity were respected. R software was used for all statistical analyses.

## 3 RESULTS

### 3.1 Initial litter characteristics

The three leaf species differed markedly in terms of their initial characteristics (Table 2). Specifically, *A. chinense* had the lowest content of nitrogen, phosphorus, and lignin, and the lowest toughness, C/N and lignin/N ratios (*p* < .05 for all). *M. leptophylla* had the highest content of carbon and lignin, and the greatest toughness and lignin/N ratio (*p* < .05 for all). *L. acalycina* had the intermediate values in terms of the content of lignin and lignin/N ratios.

**TABLE 2:** Content of carbon, nitrogen, phosphorus, and lignin; ratios of lignin:N, C:N, C:P, and N:P; and toughness of the three leaf species (mean ± *SE*; *n* = 5)

Species	C (% DM)	N (% DM)	P (% DM)	Lignin (% DM)	Lignin:*N*	C:N	C:P	N:P	Toughness (N/mm)
*Alangium chinense*	36.49 ± 0.25 ^a^	1.55 ± 0.06 ^c^	0.19 ± 0.002 ^b^	3.81 ± 0.05 ^a^	2.46 ± 0.10 ^a^	23.60 ± 1.14 ^a^	193.29 ± 2.94 ^a^	8.22 ± 0.29 ^a^	0.24 ± 0.02 ^a^
*Liquidambar acalycina*	36.34 ± 0.36 ^a^	0.99 ± 0.02 ^a^	0.10 ± 0.006 ^a^	10.20 ± 1.29 ^b^	10.29 ± 1.24 ^b^	36.74 ± 1.00 ^c^	370.10 ± 20.25 ^b^	10.10 ± 0.70 ^a^	0.26 ± 0.02 ^a^
*Machilus leptophylla*	40.08 ± 0.22 ^b^	1.22 ± 0.03 ^b^	0.10 ± 0.004 ^a^	25.52 ± 0.99 ^c^	28.89 ± 1.00 ^c^	32.82 ± 0.94 ^b^	419.15 ± 15.52 ^b^	12.82 ± 0.86 ^b^	0.35 ± 0.03 ^b^

Different letters within a column indicate significant differences among species for each parameter (one‐way ANOVA with LSD test, *p* < .05).

### 3.2 Litter decomposition rates

**FIGURE 2:**
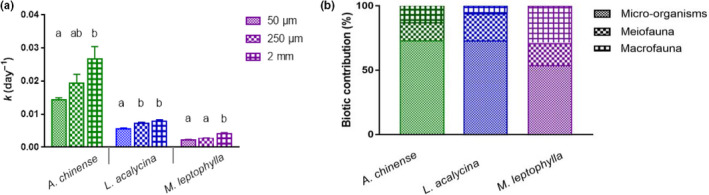
(a) Litter decomposition rates (*k*) (day^−1^) (mean ± *SE*, *n* = 4) for different mesh‐size litter bags for the three leaf species. For a given species and date, significant differences among the mesh sizes are indicated by different letters (one‐way ANOVA and LSD test, *p* < .05); (b) Contribution of different biotic communities to leaf mass loss in litter bags after the 78 days of immersion

Litter decomposition rates (*k*) varied 10‐fold (from 0.0022 to 0.033/day) (Figure 2), with significant differences between litter types (*M. leptophylla* < *L. acalycina* < *A. chinense*, *F*_2,27_ = 96.0, *p* < .001) and mesh sizes (*F*_2,27_ = 9.0, *p* < .001). The interactions between mesh size and species for the decomposition rates were significant (species × mesh, *F*_4,27_ = 3.6, *p* = .02; Table 3). The mean decomposition rates for all three litter species showed generally increasing trends with mesh size (Figure 2a), implying that the increasing community complexity enhanced the litter decomposition. The decomposition rates for all three leaves in the coarse‐mesh bags were significantly higher than those in the fine‐mesh bags (LSD test, *p* < .001). In particular, for *L. acalycina*, the decomposition rates in the fine‐mesh bags were significantly lower than those in the medium‐mesh bags (meiofauna and microorganisms present), suggesting a positive meiofaunal effect (LSD test, *p* = .02). Moreover, its decomposition rates in medium‐ and coarse‐mesh bags were not significantly different (LSD test, *p* > .05) (Figure 2a).

**TABLE 3:** Summary of ANOVA testing of the effects of leaf species and mesh size on litter decomposition rates (*k*); effects of leaf species, mesh size, and time on mass remaining and fungal biomass

	*k*			Mass remaining			Fungal biomass		
	*df*	*F*	*p*	*df*	*F*	*p*	*df*	*F*	*p*
Species	2	95.99	***	2	968.09	***	2	10.08	***
Mesh	2	9.05	***	2	31.39	***	2	17.68	***
Time				2	323.57	***	2	6.48	***
Species × mesh	4	3.62	***	4	1.91	.12	4	13.48	***
Species × time				4	32.31	***	3	25.72	***
Mesh × time				4	10.48	***	4	2.02	.100
Species × mesh ×time				8	1.58	.14	6	8.68	***
	27			81			68		

*** *p* < .001, **p < .01, *p < .05.

### 3.3 Contribution of biotic communities to litter decomposition

The relative contribution of microbes, meiofauna, and macrofauna differed among the litter species (Figure 2b). Clearly, microbes played a major role in litter decomposition, with relative contributions being 72.6% for *A. chinense*, 72.8% for *L. acalycina*, and 53.8% for *M. leptophylla*. The relative contribution of meiofauna to the mass loss for the three species was in the following order: *L. acalycina* (21.0%) > *A. chinense* (14.1%) ≈ *M. leptophylla* (13.8%). The relative contribution of macrofauna to the mass loss for the three species was in the following order: *M. leptophylla* (29%) > *A. chinense* (13.3%) > *L. acalycina* (6.2%).

### 3.4 Mass remaining

**FIGURE 3:**
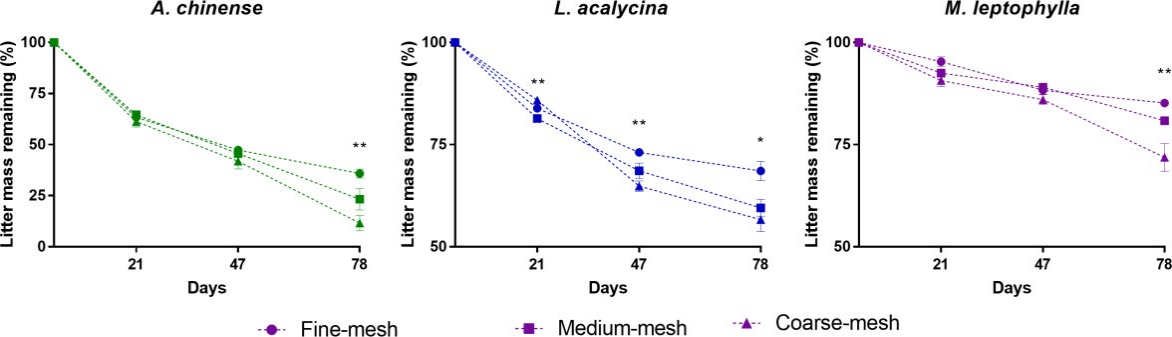
Mass remaining (%) (mean ± *SE*, *n* = 4) of different mesh‐size litter bags for the three leaf species over the experimental period. For a given species and date, significant differences among the mesh sizes are indicated by the stars (*p < .05, **p < .01, ***p < .001)

Mesh size effects for leaf remaining mass of leaves significantly differed over time (mesh × time, *F*_4,81_ = 10.5, *p* < .001). Specifically, the mesh effects on the mass loss of *A. chinense* were only significant on day 78, with the highest values in coarse‐mesh bags (*F*_2,9_ = 9.5, *p* = .006). For *L. acalycina*, on day 21, its mass loss significantly increased (coarse‐mesh > fine‐mesh > medium‐mesh, LSD test, *p* < .05), while on days 47 and 78, the mass losses in medium‐mesh were significantly greater than those in fine‐mesh bags (LSD test, *p* = .05, *p* = .03) and no significant difference was detected between the mass loss in coarse‐ and medium‐mesh bags (LSD test, *p* > .05). For *M. leptophylla*, the mass loss in coarse‐mesh bags was significantly greater than that in fine‐mesh bags on day 78 (LSD test, *p* = .001), while the mass losses in fine‐ and medium‐mesh bags were not significantly different (LSD test, *p* > .05; Figure 3).

### 3.5 Fungi

**FIGURE 4:**
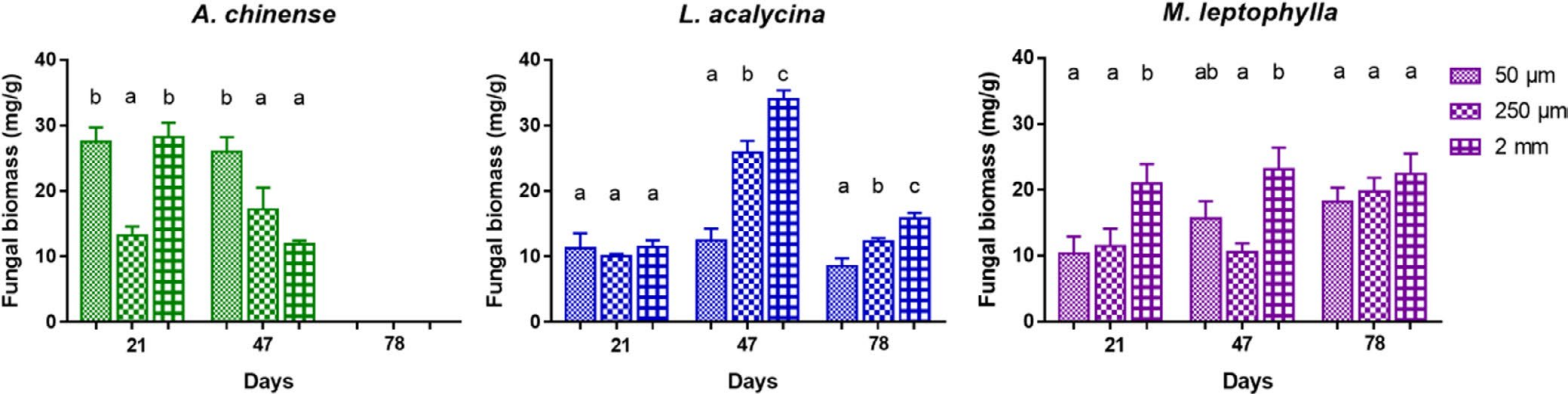
Fungal biomass (mg/g leaf DM) (mean ± *SE*, *n* = 4) in litter bags of three mesh sizes for the three leaf species over the experimental period. For a given species and date, significant differences among mesh size are indicated by different letters (one‐way ANOVA and LSD test, *p* < .05)

Fungal biomass fluctuated over time and differed across species and mesh sizes (Table 3, Figure 4). *A. chinense* supported the highest fungal biomass among the species (*F*_2,68_ = 10.1, *p* < .001). The fungal biomass was the significantly higher on day 47 than on other dates (*F*_2,68_ = 6.5, *p* < .01). Specifically, for a given species and date, the associated fungal biomass for *A. chinense* in medium‐ and coarse‐mesh bags was significantly lower than for fine‐mesh bags on day 47 (LSD test, *p* = .03, *p* = .002). For *L. acalycina*, the associate fungal biomass significantly increased with mesh size on day 47, by onefold for medium‐mesh bags and twofold for coarse‐mesh bags compared to fine‐mesh (fine‐mesh < medium‐mesh < coarse‐mesh, LSD test, *p* < .01). This increasing trend was still seen day 78 (fine‐mesh < medium‐mesh < coarse‐mesh, LSD test, *p* < .05). For *M. leptophylla*, the associated fungal biomass peaked in the coarse‐mesh on days 21 and 47 (*F*_2,29_ = 4.5, *p* = .04; *F*_2,29_ = 6.0, *p* = .02), though it did not significantly differ among mesh types on day 78 (*F*_2,29_ = 0.7, *p* > .05; Figure 4).

**FIGURE 5:**
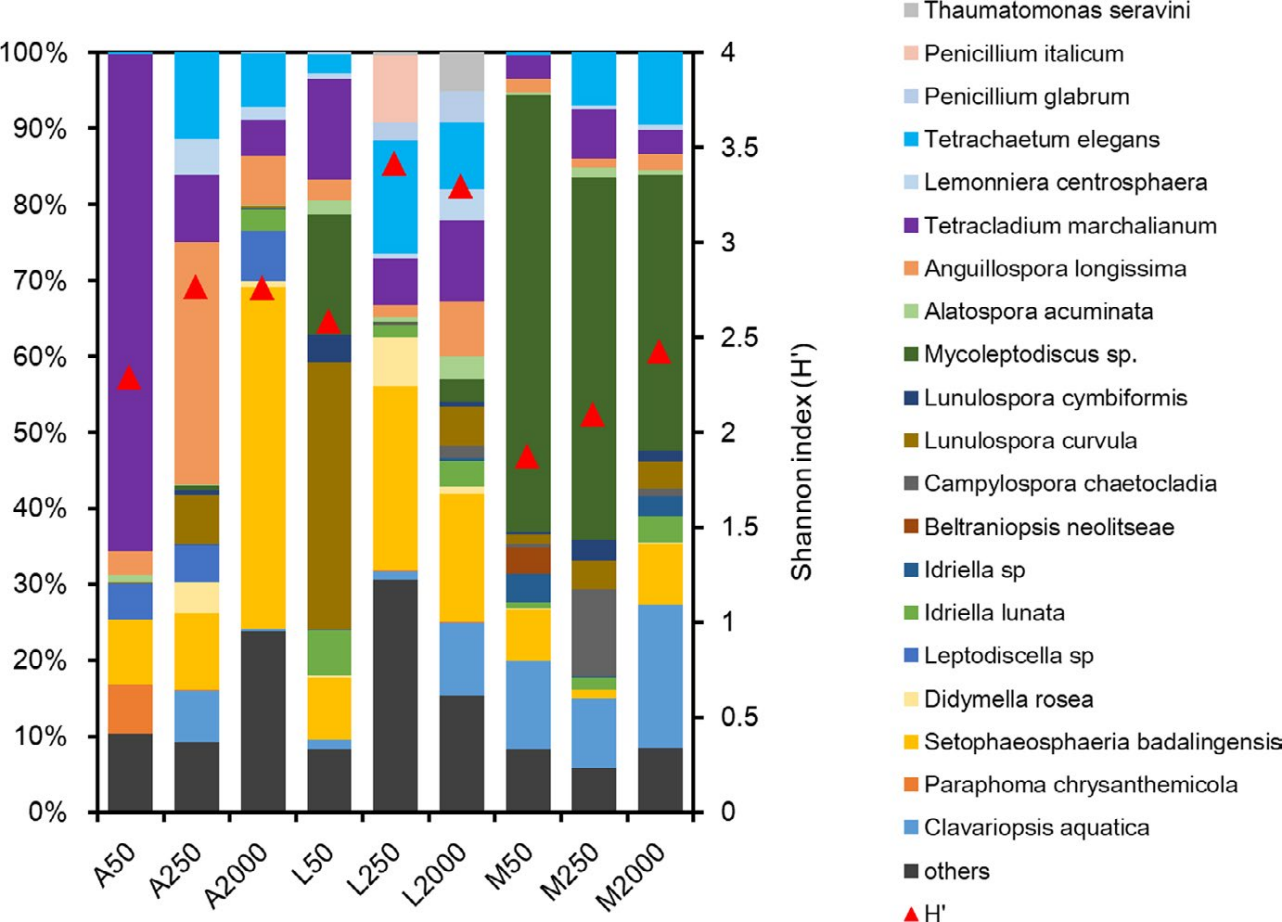
Relative abundance of fungal genera (>3.0%) in litter bags of three mesh sizes for the three litter species (For labels on the *x*‐axis: A: *Alangium chinense*, L: *Liquidambar acalycina*, M: *Machilus leptophylla*; 50:50 µm, 250:250 µm, 2,000:2,000 µm). Triangles indicate the Shannon index (H′) of the fungal community

A total of 55,846 OTUs were found based on ITS gene sequencing. The fungal community on leaf material was dominated by taxa belonging mainly to the Ascomycota phyla, which made up 97.5% of all recorded OTUs, followed by Rozellomycota (0.6%) and Cercozoa (0.6%). Fungal assemblages depended on the leaf species (Figure 5). Specifically, in the fine‐mesh bags, *Tetracladium marchalianum* was the dominant species on *A. chinense* (64.8%), *Lunulospora curvula* (35.2%) dominated on *L. acalycina*, and *Mycoleptodiscus* sp. (57.4%) dominated on *M. leptophylla*. Fungal composition, and their diversity and abundance were modified by the presence of meio‐ and macrofauna in medium‐ and coarse‐mesh bags, respectively. In particular, for *A. chinense*, fungi *Anguillospora longissima* mostly contributed to the total fungal abundance (31.9%) in medium‐mesh bags, while *Setophaeosphaeria badalingensis* was the dominant fungal species in coarse‐mesh bags (Figure 5). The fungal diversity for *A. chinense* increased with increasing mesh size, but the fungal abundance was lowest in the coarse‐mesh bags (Figure 7). For *L. acalycina*, the relative abundance of *S. badalingensis* and *Tetrachaetum elegans* increased in medium‐ and coarse‐mesh bags, while the relative abundance of *L. cymbiformis* decreased with increasing mesh size. Higher fungal diversity and abundance were found in medium‐ and coarse‐mesh bags, in comparison with the fine‐mesh bags (Figures 5 and 7). For the leaf *M. leptophylla*, *Mycoleptodiscus* sp. was the dominant group in the different mesh sizes, but its proportion decreased (57.4% for fine‐mesh, 47.5% for medium‐mesh, and 36.2% for coarse‐mesh bags), which was accompanied by increasing fungal diversity with increasing mesh sizes (1.87 for fine‐mesh, 2.1 for medium‐mesh, and 2.4 for coarse‐mesh bags; Figures 5 and 7).

### 3.6 Bacteria

**FIGURE 6:**
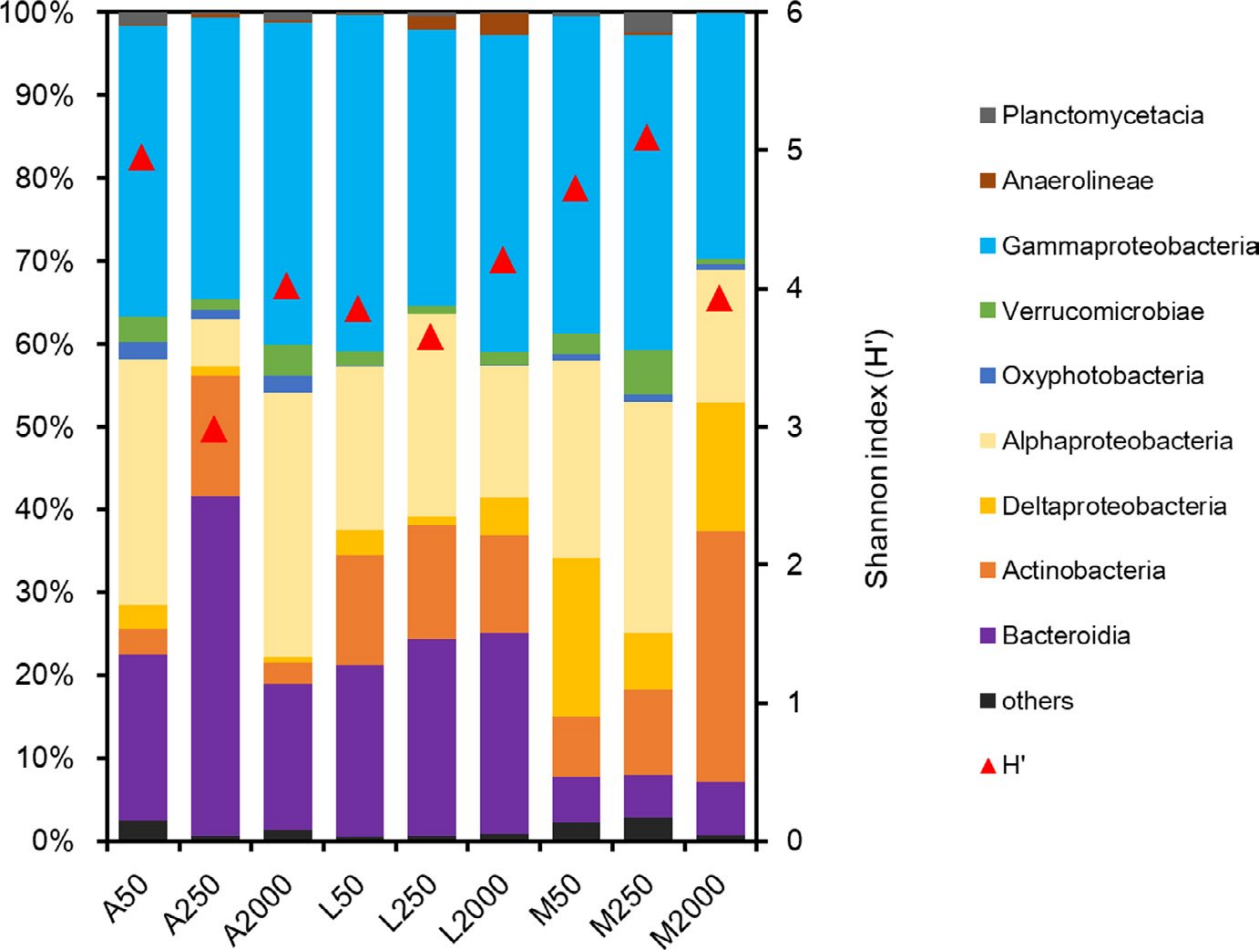
Relative abundance of bacterial classes (>1.0%) in litter bags of three mesh sizes for the three litter species (For labels on the *x*‐axis: A: *Alangium chinense*, L: *Liquidambar acalycina*, M: *Machilus leptophylla*; 50:50: 50 µm, 250:250 µm, 2,000:2,000 µm). Triangles indicate the Shannon index (H′) for the bacterial community

**FIGURE 7:**
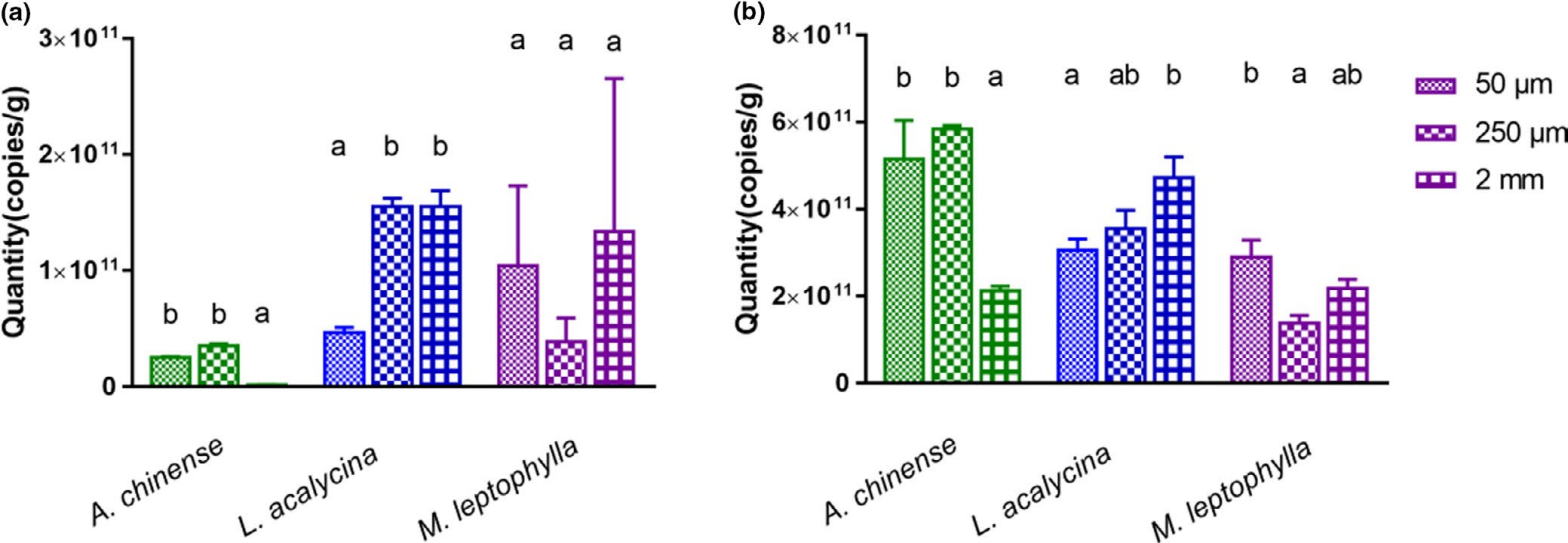
Abundance of fungal ITS gene (a) and bacteria 16S gene (b) in litter bags of three mesh sizes for the three litters species (the number of copies per gram of leaf DM, measured using qPCR). For each species, significant differences among the mesh sizes are indicated by different letters (one‐way ANOVA and LSD test, *p* < .05)

A total of 41,794 OTUs were found based on sequence analyses of the leaf‐associated bacterial 16S rRNA gene pool. The Proteobacteria were found to be the major bacteria phylum present in the litter bags (64.05) with γ‐Proteobacteria being the most abundant class for all three leaves and mesh sizes (Figure 6). In addition, the bacterial communities on *A. chinense* and *L. acalycina* contained a large proportion of Bacteroidia class, which were in low abundance on *M. leptophylla*. Actinobacteria and α‐Proteobacteria were the main groups on *M. leptophylla*. The bacterial diversity and abundance for the three leaves fluctuated among the different mesh types (Figures 6 and 7). The highest bacterial diversity for *A. chinense* was found in the fine‐mesh bags, while the highest bacterial diversity for *L. acalycina* was found in the coarse‐mesh bags, and for *M. leptophylla*, it was found in the medium‐mesh bags (Figure 6).

### 3.7 Meiofauna and macrofauna

A total of 16 meiofaunal taxa were found with a mean density of 9,529 ± 2,658 ind./m^2^ during the whole study (Table S1). With a relative abundance of 59.2%, meiobenthic Chironomidae larvae were the most abundant organisms, followed by nematodes (26.5%) and meiobenthic Ephemeroptera (5.6%). The largest number of meiofauna was collected in the middle stage with a mean density of 16,256 ± 6,499 ind./m^2^, while the smallest number was found in the last collection with a mean density of 2,561 ± 658 ind./m^2^. At the end of the experiments, meiobenthic Chironomidae larvae remained dominant (58.8%) and the percentage of Ostracoda increased to 15.1% (Table S1). A total of 32 macrofaunal taxa were identified with a mean density of 2,072 ± 396 ind./m^2^ during the experimental period (Table S2). On average, Ephemeroptera accounted for most of the total macrofaunal density (39.7%), followed by Diptera (33.4%), Coleoptera (18.0%), Plecoptera (6.1%), and a few Acariformes (2%). The dominant functional groups of macrofauna were scrapers (49.1%), which were followed by shredders (18.2%), deposit feeders (17.5%), predators (9.9%), filter feeders (5.2%), and piercers (0.1%). Heptageniidae, Elmidae, Chloroperlidae, and Chironomidae (Cladotanytarsus) were the main groups that were shredding leaves (Table S2).

## 4 DISCUSSION

### 4.1 Effect of initial litter quality

The different decomposition rates across leaf species may be mainly due to their initial chemical characteristics (Ágoston‐Szabó et al., 2016; Bruder, Schindler, Moretti, & Gessner, 2014). In general, the contents of C, N, P elements and lignin in leaves are important factors affecting decomposition rates (Santschi, Gounand, Harvey, & Altermatt, 2018). The relative order of decomposition rate among the three leaf types was *A. chinense* > *L. acalycina* > *M. leptophylla*. Reversed orders were found for the content of lignin and the lignin/N ratio, and the lignin/N ratio was used to classified litter quality as high‐(*A. chinense*), medium‐(*L. acalycina*), or low‐(*M. leptophylla*) quality species.

The three leaf species could be classified into different decomposition groups according to the decomposition rates in temperate streams(Bruder et al., 2014; Ferreira & Canhoto, 2015; Petersen & Cummins, 1974): *A. chinense* (fast) > *L. acalycina* (medium) > *M. leptophylla* (slow). *L. acalycina* and *M. leptophylla* decomposed more slowly than similar species in tropical regions (i.e.,* Liquidambar formosana* and *Ocotea* sp.) (Li, Ng, & Dudgeon, 2009; Ligeiro, Moretti, Gonc, & Callisto, 2010), probably because of the relatively higher temperature in tropical regions (Li et al., 2009). In particular, the decomposition of *A. chinense* was first measured in the subtropical region of China. The decomposition rates of the three species in this study could help to expand the models of leaf resistivity to decomposition in subtropical regions (i.e., from the most labile *A. chinense* to the most recalcitrant *M. leptophylla* tree species).

### 4.2 Relative contributions of biotic communities

In this work, microbes were major contributors to litter decomposition. Fungi, especially aquatic hyphomycetes, such as *T. elegans*, *T. marchalianum*, and *L. curvula*, are known to play crucial roles in litter decomposition (Graça, Hyde, & Chauvet, 2016; Seena et al., 2019). In addition, some of the major bacteria groups that are known to participate in litter decomposition were also observed, such as Actinobacteria (order Micromonospora; Wohl & McArthur, 1998), α‐Proteobacteria (order Sphingomonadales) (Newman, Liles, & Feminella, 2015), γ‐Proteobacteria (order Pseudomonadales; Xu et al., 2013), and Bacteroidia (order Flavobacteriales) (Zhao, Xing, & Wu, 2017). In contrast, invertebrates contributed a relatively small amount to the litter decomposition here, in agreement with the reports for other tropical and subtropical ecosystems (Al‐Riyami, Victor, Seena, Elshafie, & Bärlocher, 2009; Boyero et al., 2011; Graça et al., 2016). This may be explained by that common shredders in temperate regions (e.g., Trichoptera and Amphipoda) were lacking in this subtropical ecosystem while scrapers were the dominate functional groups in this study (Graça et al., 2016; Tanaka, Ribas, & de Souza, 2006).

The presence of meiofauna and macrofauna significantly increased the leaf decomposition rates, when compared to a single microbial treatment. This suggests the crucial role played by trophic complexity with regard to litter decomposition in decomposer communities (Santschi et al., 2018; Stocker et al., 2017). The effect of invertebrates on the decomposition process became increasingly important with decreasing leaf quality. High‐quality leaf species are important carbon sources for microbial consumers, while low‐quality species are important for invertebrates as substrata for attachment and eventually as a source of particulate organic matter (Ardón & Pringle, 2008).

### 4.3 Effect of meiofauna

In this study, meiofauna mainly included early‐stage chironomid larvae and nematodes, with the former accounting for about 60% of the total meiofaunal density. These dominant meiofaunal species also described by other authors (Gaudes, Artigas, & Muñoz, 2010; Majdi, Boiché, Traunspurger, & Lecerf, 2015). Density found in this stream was in the range reported in other streams (Gaudes et al., 2010; Swan & Palmer, 2000).

The meiofauna can affect litter decomposition in several key ways: (a) as microshredders for the direct consumption of leaves; (b) grazing and selective feeding on fungal communities; and (c) bioturbation effects on the leaf‐associated microenvironment (Mathieu, Leflaive, Ten‐Hage, De Wit, & Buffan‐Dubau, 2007; Perlmutter & Meyer, 1991; Traunspurger, Bergtold, & Goedkoop, 1997). In this study, the meiofauna significantly improved the decomposition rates of *A. chinense* and *L. acalycina*, rather than on *M. leptophylla*.

Regarding the low‐quality species (*M. leptophylla*), the meiofauna significantly affected neither the leaf decomposition nor the associated fungal biomass. Compared to the macrofauna, the meiofauna had a reduced direct consumption of low‐quality leaves (Nolen & Pearson, 1993).

The presence of meiofauna significantly enhanced the decomposition of high‐ and medium‐quality leaves, but in different ways. Specifically, the meiofauna's grazing effect decreased the fungal biomass for high‐quality leaves but increased the fungal biomass and diversity of medium‐quality leaves. A positive relationship was seen between fungal biomass and litter decomposition, so that a reduced fungal biomass may have led to the slow litter decomposition (Duarte, Pascoal, Alves, Correia, & Cássio, 2010; Pascoal & Cássio, 2004). The top‐down effect of the small chironomid larvae on fungal biomass and respiratory activity were also found for *Salix alba*, another high‐quality leaf species (Ágoston‐Szabó et al., 2016). Nevertheless, the meiofauna stimulated fungal biomass of medium‐quality leaves, probably because the moderate grazing on aging microorganisms can keep the fungal community active and increase the fungal demand for nutrients from leaves (Chen & Wang, 2018; Lillebø, Flindt, Pardal, & Marques, 1999; Piot, Nozais, & Archambault, 2013). Taking together, for high‐quality leaves, the direct consumption by meiofauna outweighed the potential negative effect on decomposition caused by the reduced fungal biomass. Moreover, for medium‐quality leaves, (a) direct consumption, (b) facilitation of fungal biomass development, and (c) leaf surface bioturbation caused by the meiofauna likely contributed to the mutual, nonexclusive stimulation of litter composition. Our results show that as the quality of leaves decreases, the meiofauna can modulate microbial activity by suppressing or facilitating the fungal biomass. This resulted in a negative, positive, or neutral interaction between microbes and meiofauna for high‐, medium‐, and low‐quality species respectively.

These findings, with regard to the meiofauna, were the leaf quality dependent. Different qualities of leaf type support different microbial communities (Gulis, 2001; Jabiol & Chauvet, 2012) and may also support different colonized meiofauna. Meanwhile, the meiofauna can change the fungal composition by preferentially feeding on some taxa. For instance, fungivorous nematodes (e.g., from the Aphelenchida) have very specific fungal diets (Dighton, Zapata, & Ruess, 2000). The selective feeding of meiofauna could reduce the growth of some fungi taxa, while promoting others, thereby reducing the competition for resources between different fungi species (Chambord et al., 2017). Indeed, the meiofauna reduced the relative abundance of *Alatospora acuminata* and *T. Marchalianum* but increased *T. elegans* and *Penicillium italicum* that showed specific ligninolytic activities (Hofmann et al., 2016; Osono, 2007). Because of the complexity and importance of the feeding preference for meiofauna, like those of the shredding macroinvertebrates, further studies are warranted (Canhoto & Graça, 2008; Mora‐Gómez et al., 2016). Moreover, the competition between fungi and bacteria may also contribute to changes in the fungal community composition. The consumption of bacteria by meiofauna may stimulate fungal growth by decreasing the competition, thus favoring changes in the fungal community composition (Chambord et al., 2017). The meiofauna altered fungal biomass and composition may further change fungal sporulation and enzyme activities (Mora‐Gómez et al., 2016), resulting in different microbial contributions to the decomposition of litter of different quality (Jabiol & Chauvet, 2012).

### 4.4 Effect of macrofauna

The presence of macrofauna increased the effect of the meiofauna on the decomposition of leaves. Shredders accounted for about 20% of the total macrofaunal density, indicating the direct consumption of leaves. Then, the indirect effect of macrofauna on litter decomposition by grazing microbes and bioturbating the microenvironment could be also expected (Mora‐Gómez et al., 2016; Solan, Batty, Bulling, & Godbold, 2008; Villanueva et al., 2012). In addition, since predators such as Hydrachnidia were found, the macrofauna may have a top‐down control effect on the meiofauna.

Specifically, for high‐quality leaf species, the presence of macrofauna reduced the fungal biomass compared to that in the fine‐mesh bags during the mid‐decomposition period. Nevertheless, their direct consumption of leaves outweighed the negative effects of the grazing on fungi, finally enhancing the litter decomposition of high‐quality leaves.

For medium‐quality leaf species, the macrofauna could maintain the positive interactions between meiofauna and leaf‐associated fungi, though this may have been offset by the predation of macrofauna on the meiofauna (Ptatscheck et al., 2020).

For low‐quality leaf species, the role of the macrofauna is even more pronounced. The macrofauna's direct consumption produced fragments of plant material that could allow microorganisms to enter the nutritious internal tissues. The fragments can be more readily utilized by meiofauna and microbes, favoring decomposition (Santonja et al., 2018). In addition, the increased abundance and diversity of microbes in the presence of macrofauna may also contribute to decomposition by improving the palatability of leaves for the macrofauna.

## 5 CONCLUSION

With regard to increased trophic complexity, this study provides evidence that the presence of meiofauna and meiofauna + macrofauna promotes the decomposition of three litter species having different chemical qualities.

Our results show that the meiofauna significantly contributed to the detrital process in aquatic ecosystems. For a high‐quality species, the meiofauna promoted the litter decomposition mainly through direct consumption, although they had the negative interactions with microbes. For a medium‐quality species, besides direct consumption, the positive interaction between meiofauna and microbes also promoted litter decomposition. For a low‐quality species, litter decomposition was not significantly promoted by meiofauna but decomposition was increased by a combination of macrofauna and meiofauna. For any leaf species, macrofauna systematically increased the effect of meiofauna on leaf decomposition. The presence of meiofauna and macrofauna triggered different microbial communities, with effects on litter decomposition that varied with leaf quality.

Overall, for detritus‐based ecosystems, these findings imply that the meiofauna and trophic diversity in the decomposer community are crucial for litter decomposition and subsequent nutrients dynamics.

## CONFLICT OF INTEREST

The authors declare that they have no known competing financial interests or personal relationships that could have appeared to influence the work reported in this paper.

## AUTHOR CONTRIBUTION

**Fang Wang:** Data curation (lead); Investigation (lead); Methodology (supporting); Software (lead); Writing‐original draft (lead); Writing‐review & editing (supporting). **Dunmei Lin:** Conceptualization (supporting); Investigation (supporting); Resources (equal); Writing‐review & editing (supporting). **Wei Li:** Investigation (supporting); Methodology (supporting); Supervision (supporting); Validation (supporting); Writing‐original draft (supporting); Writing‐review & editing (supporting). **Pengpeng Dou:** Investigation (supporting); Methodology (supporting). **Le Han:** Validation (supporting); Writing‐original draft (supporting); Writing‐review & editing (supporting). **Mingfen Huang:** Data curation (supporting); Investigation (supporting). **Shenhua Qian:** Software (supporting); Validation (supporting); Writing‐review & editing (supporting). **Jingmei Yao:** Conceptualization (lead); Funding acquisition (lead); Investigation (lead); Methodology (lead); Project administration (lead); Resources (equal); Supervision (lead); Writing‐original draft (lead); Writing‐review & editing (lead).

## Supporting information

Table S1‐S2

## Data Availability

The data that support the findings of this study are openly available in the Dryad Digital Repository at http://doi.org/10.5061/dryad.cfxpnvx3g.
