# Supplementary material for: Meiofauna promotes litter decomposition in stream ecosystems depending on leaf species
Source: Ecol Evol. 2020 Aug 3;10(17):9257–70. doi: 10.1002/ece3.6610 (PMC7487239; doi:10.1002/ece3.6610)
Supplement: Supplementary file 1 — Table S1‐S2 [file ECE3-10-9257-s001.docx]

Table S1. Taxonomic and functional feeding group (FFG) mean (± SE) density (ind./m^2^) and total percentage (% total) of aquatic meiofauna in the study site over the experimental period. DF: Deposit feeder, SH: Shredder, SC: Scrapers, F: Filter feeder, PI: Piercer, PR: Predator, P: Parasite.

|  | FFG | Density (ind./m^2^) | | | | Total |
| --- | --- | --- | --- | --- | --- | --- |
|  |  | 0d | 21d | 47d | 78d | (%) |
| **Ostracoda** | F | 534±271.6 | 111±12.7 | 450±157.0 | 673±234.1 | **4.30** |
| **Nematode** | DF/SH/P | 2237±2060.0 | 3889±2219.6 | 3777±1411.3 | 978±352.8 | **26.46** |
| **Copepoda** |  |  |  |  |  | **2.14** |
| Harpacticoida | DF | 207±103.8 | 245±89.8 | 245±89.8 | 106±41.9 | 1.95 |
| Cyclopoida | DF | 0±0.0 | 0±0.0 | 78±45.0 | 0±0.0 | 0.19 |
| **Oligochaeta** | DF/SC | 15±14.7 | 22±12.7 | 22±12.7 | 33±13.0 | **0.22** |
| **Coleoptera** |  |  |  |  |  | **0.22** |
| Elmidae |  |  |  |  |  |  |
| *Limmius* | SC/SH | 67±38.4 | 0±0.0 | 11±6.4 | 0±0.0 | 0.19 |
| Dytiscidae |  |  |  |  |  |  |
| *Laccophilus* | SH/PI | 15±14.7 | 0±0.0 | 0±0.0 | 0±0.0 | 0.03 |
| **Ephemeroptera** |  |  |  |  |  | **5.63** |
| Ephemeridae |  |  |  |  |  |  |
| *Ephemera* |  |  |  |  |  |  |
| *Ephemera orientalis* | SH/F/DF/PR | 0±0.0 | 22±12.7 | 0±0.0 | 0±0.0 | 0.05 |
| Baetidae |  |  |  |  |  |  |
| *Baetis* | SC/DF | 370±148.3 | 0±0.0 | 0±0.0 | 0±0.0 | 0.90 |
| Heptageniidae |  |  |  |  |  |  |
| *Cinygmina* | SC | 222±92.5 | 111±64.1 | 134±25.7 | 222±92.5 | 1.14 |
| *Iron* | SC | 570±410.0 | 0±0.0 | 861±298.5 | 0±0.0 | 3.48 |
| *Heptagenia* | SC/SH | 0±0.0 | 0±0.0 | 0±0.0 | 22±12.7 | 0.05 |
| **Plecoptera** |  |  |  |  |  | **0.18** |
| Chloroperlidae |  |  |  |  |  |  |
| *Siphonoperla* | SH | 52±41.2 | 22±12.7 | 0±0.0 | 0±0.0 | 0.18 |
| **Diptera** |  |  |  |  |  | **59.20** |
| Chironmidae | SC/DF/PR | 4674±2841.0 | 3666±1706.4 | 10378±4837.0 | 2561±657.6 | 59.05 |
| Cetatqogoridae | PR/DF/SH | 0±0.0 | 0±0.0 | 0±0.0 | 61±22.5 | 0.15 |
| **Acariformes** |  |  |  |  |  | **1.64** |
| Hydrachnidia | PR | 260±57.9 | 88±25.7 | 300±32.3 | 28±16.2 | 1.64 |

Table S2. Taxonomic and functional feeding group (FFG) mean (± SE) density (ind./m^2^) and total percentage (% total) of aquatic macrofauna in the study site over the experimental period. DF: Deposit feeder, SH: Shredder, SC: Scrapers, F: Filter feeder, PI: Piercer, PR: Predator, P: Parasite.

|  | FFG | Density (ind./m^2^) | | | | Total |
| --- | --- | --- | --- | --- | --- | --- |
|  |  | 0d | 21d | 47d | 78d | (%) |
| **Coleoptera** |  |  |  |  |  | **18.02** |
| Elmidae |  |  |  |  |  |  |
| *Limmius* | SC/SH | 393±70.8 | 245±33.5 | 337±115.7 | 281±105.6 | 15.45 |
| *Macronychus* | SC/SH | 0±0.0 | 0±0.0 | 15±9.7 | 4±3.7 | 0.24 |
| *Elmis* | SC/SH | 0±0.0 | 17±16.5 | 0±0.0 | 0±0.0 | 0.14 |
| *Esolus* | SC/SH | 0±0.0 | 0±0.0 | 11±11.0 | 48±31.7 | 0.78 |
| Elmidae | SC/SH | 0±0.0 | 0±0.0 | 48±42.6 | 15±9.7 | 0.82 |
| Psephenidae | SC | 4±3.7 | 0±0.0 | 11±11.0 | 11±11.0 | 0.34 |
| Dytiscidae |  |  |  |  |  |  |
| *Laccophilus* | SH/PI | 15±7.3 | 0±0.0 | 0±0.0 | 4±3.7 | 0.24 |
| **Ephemeroptera** |  |  |  |  |  | **39.72** |
| Ephemeridae |  |  |  |  |  |  |
| *Ephemera* |  |  |  |  |  |  |
| *Ephemera orientalis* | SH/F | 82±71.0 | 22±0.0 | 0±0.0 | 15±9.7 | 1.46 |
| Baetidae |  |  |  |  |  |  |
| *Baetis* | SC/DF | 7±7.3 | 122±100.0 | 656±101.7 | 1326±988.2 | 27.25 |
| Heptageniidae |  |  |  |  |  |  |
| *Cinygmina* | SC | 96±90.9 | 6±5.5 | 55±29.4 | 115±49.1 | 3.55 |
| *Ecdyonurus* |  |  |  |  |  |  |
| *Ecdyonurus dracon* | SC/SH | 63±42.6 | 39±39.0 | 196±74.7 | 230±104.4 | 6.77 |
| Neoephemeridae |  |  |  |  |  |  |
| *Neoephemera* | DF | 0±0.0 | 0±0.0 | 0±0.0 | 52±41.2 | 0.68 |
| **Plecoptera** |  |  |  |  |  | **6.13** |
| Chloroperlidae |  |  |  |  |  |  |
| *Siphonoperla* | SH | 41±40.7 | 95±72.5 | 174±89.8 | 18±13.2 | 3.90 |
| Perlidae |  |  |  |  |  |  |
| *Dinocras* | SH/PR | 0±0.0 | 0±0.0 | 26±3.7 | 7±3.7 | 0.43 |
| Capniidae |  |  |  |  |  |  |
| *Capnia* | SH | 0±0.0 | 0±0.0 | 4±3.7 | 41±35.3 | 0.58 |
| *Capnioneura* | SH | 0±0.0 | 28±28.0 | 48±18.7 | 26±13.2 | 1.22 |
| **Diptera** |  |  |  |  |  | **33.41** |
| Tipulidae | SH/DF/PR | 0±0.0 | 11±11.0 | 15±9.7 | 26±3.7 | 0.63 |
| Limoniidae | SH/SC/PR | 29±14.7 | 50±6.0 | 56±23.2 | 37±10.0 | 2.04 |
| Chironmidae |  |  |  |  |  |  |
| Tanypodinae |  |  |  |  |  |  |
| *Paramerina* | PR | 0±0.0 | 139±83.0 | 148±102.2 | 56±29.4 | 3.90 |
| Tanytarsini |  |  |  |  |  |  |
| *Cladotanytarsus* | DF/SH/SC | 192±99.5 | 167±11.0 | 481±265.7 | 96±96.3 | 11.60 |
| *Tanytarsus* | DF/SH/SC | 22±11.0 | 22±0.0 | 55±33.9 | 63±63.0 | 2.04 |
| Chironomini |  |  |  |  |  |  |
| *Polypedilum* | DF/SH/F | 93±16.0 | 56±0.0 | 167±97.3 | 96±96.3 | 5.17 |
| Orthocladiinae |  |  |  |  |  |  |
| *Eukiefferiella* | SC/DF/F | 33±17.0 | 28±5.5 | 85±47.4 | 48±48.0 | 2.44 |
| *Heleniella* | SC/DF/F | 22±11.0 | 22±0.0 | 52±30.3 | 33±33.3 | 1.60 |
| *Parachaetocladius* | SC/DF/F | 11±6.4 | 11±0.0 | 26±16.3 | 15±14.7 | 0.78 |
| *Rheocricotopus* | SC/DF/F | 11±6.4 | 11±0.0 | 26±16.3 | 15±14.7 | 0.78 |
| Cetatqogoridae | PR/DF/SH | 0±0.0 | 22±22.0 | 78±23.2 | 81±35.3 | 2.29 |
| Empididae | SH | 11±11.0 | 0±0.0 | 0±0.0 | 0±0.0 | 0.14 |
| **Trichoptera** |  |  |  |  |  | **0.14** |
| Polycentropodidae | PR/F | 0±0.0 | 0±0.0 | 0±0.0 | 11±6.4 | 0.14 |
| **Acariformes** |  |  |  |  |  | **2.14** |
| Hydrachnidia | PR | 66±39.0 | 72±61.0 | 44±11.3 | 4±3.7 | 2.14 |
| **Basommatophora** |  |  |  |  |  | **0.43** |
| Hydrobiidae | SC/SH/F | 11±0.0 | 11±0.0 | 0±0.0 | 4±3.7 | 0.29 |
| Planorbidae | SC/SH | 0±0.0 | 0±0.0 | 7±7.3 | 4±3.7 | 0.14 |
